# Effect of Hydrogel Contact Angle on Wall Thickness of Artificial Blood Vessel

**DOI:** 10.3390/ijms231911114

**Published:** 2022-09-21

**Authors:** Wenyu Jin, Huanbao Liu, Zihan Li, Ping Nie, Guangxi Zhao, Xiang Cheng, Guangming Zheng, Xianhai Yang

**Affiliations:** 1 College of Mechanical Engineering, Shandong University of Technology, Zibo 255000, China; 2 Shandong Provincial Key Laboratory of Precision Manufacturing and Non-Traditional Machining, Zibo 255000, China

**Keywords:** artificial blood vessel, 3D bioprinting, biofabrication, hydrogel, contact angle

## Abstract

Vascular replacement is one of the most effective tools to solve cardiovascular diseases, but due to the limitations of autologous transplantation, size mismatch, etc., the blood vessels for replacement are often in short supply. The emergence of artificial blood vessels with 3D bioprinting has been expected to solve this problem. Blood vessel prosthesis plays an important role in the field of cardiovascular medical materials. However, a small-diameter blood vessel prosthesis (diameter < 6 mm) is still unable to achieve wide clinical application. In this paper, a response surface analysis was firstly utilized to obtain the relationship between the contact angle and the gelatin/sodium alginate mixed hydrogel solution at different temperatures and mass percentages. Then, the self-developed 3D bioprinter was used to obtain the optimal printing spacing under different conditions through row spacing, printing, and verifying the relationship between the contact angle and the printing thickness. Finally, the relationship between the blood vessel wall thickness and the contact angle was obtained by biofabrication with 3D bioprinting, which can also confirm the controllability of the vascular membrane thickness molding. It lays a foundation for the following study of the small caliber blood vessel printing molding experiment.

## 1. Introduction

Cardiovascular diseases (CVDs) have long been the most common cause of mortality and the leading cause of morbidity worldwide. CVDs are responsible for approximately 17.9 million deaths annually, accounting for 31% of all deaths globally [1]. With the emergence of the novel coronavirus, patients with CVDs are facing great challenges. A study in the United States showed a significantly increased risk of cardiovascular disease, including cerebrovascular disease, arrhythmias, ischemic and nonischemic heart disease, pericarditis, myocarditis, heart failure, and thromboembolic disease, in patients 30 days after infection with the novel coronavirus. The risk of these cardiovascular diseases was significantly increased, even in patients with minor illnesses who were not hospitalized. The risk of developing cardiovascular disease (including heart disease and stroke) within one year remains substantially increased in those who recover after a neo-coronavirus infection, and more importantly, the risk of developing cardiovascular diseases after a neo-coronavirus infection remains significantly higher, even in those under 65 years of age who do not have risk factors for cardiovascular diseases, such as obesity, smoking, or diabetes [2]. It is foreseeable that an artificial blood vessel reserve will be very important in the coming years.

Most vessels in the cardiovascular and peripheral vascular systems are less than 6 mm in diameter. Because of the small inner diameter and slow blood flow of these vessels, the built-in scaffolds often cause problems, such as blood clots. Direct vascular prosthesis replacement is a promising approach [3]. Although a large number of vascular scaffolds have been used in clinical practice, the treatment of small diameter vascular lesions and functional defects is still a challenge because of the large size of the scaffold products [4]. In recent years, one of the research hotspots in the field of blood vessel regeneration is that an artificial blood vessel has good mechanical properties, high compliance, remodeling ability, and anti-thrombotic ability to achieve the successful combination with an autologous blood vessel, so as to achieve the basic requirements of long-term patency [5]. Three-dimensional bioprinting methods mainly include inkjet printing, extrusion printing, laser-assisted printing, and reductive polymeric bioprinting. Extrusion bioprinting is the most commonly used bioprinting method because it is easy to control and compatible with a variety of high-viscosity bioprinting inks [6,7]. Hydrogels have attracted great attention in the production of 3D bioprinting bioinks due to their good biocompatibility, high moisture content, and highly controllable 3D structure [8]. Currently, hydrogel materials that have been commonly used in 3D bioprinting include gelatin [9,10], sodium alginate [11,12], chitosan [13], agarose [14], fibrin [15], etc.

Gelatin, the most common biomaterial used to formulate hydrogel bioinks, is developed from the hydrolysis of collagen, is a common ingredient in foods, and is a source of natural protein [16]. Due to its good biocompatibility and low cost, it has been extensively studied in the biomedical field. Gelatin is a thermosensitive material that is a solid at low temperatures and gradually becomes a liquid with increasing temperature. Therefore, it is an excellent candidate for constructing different types of scaffold shapes using additive manufacturing techniques [17]. Sodium alginate is a natural polysaccharide mainly extracted from brown algae. Alginate has excellent printability and (1) good gelation characteristics, which can cross-link with calcium ions to form a gel; (2) its printability can adjust the viscosity of the solution by changing the material ratio and temperature to meet the basic requirements of extrusion printing [18].

The combination of gelatin and sodium alginate has been extensively attempted due to its properties of promoting cell proliferation and immunity [19,20,21]. Curti F. et al. [22] used a high concentration of aqueous gelatin and large incorporation of alginate to promote high-precision printing suitability and affect the in vitro stability and mechanical properties of the printed scaffolds. Han X. et al. [23] designed and prepared biohydrogels with a low elastic modulus and good formability using gelatin and sodium alginate, which exhibited good mobility before curing and maintained a specific structure after extrusion. It provided a suitable hydrogel scaffold for cell proliferation, migration, and differentiation in tissue engineering. Liu H. et al. [24] used sodium alginate and gelatin as experimental materials to fabricate multilayer vascular structures and analyzed cell loading materials, and the cell survival rate was more than 90%, which provided support for the research of printing multilayer vascular structures. Iranmanesh P. et al. [25] prepared a novel alginate/gelatin scaffold with a drug-coated surface, which can improve mechanical and biological properties.

In this paper, mixed hydrogel solutions with different concentration gradients were prepared from gelatin and sodium alginate. By measuring the contact angle of different concentrations of the hydrogel at different temperatures, the relationship between the contact angle and the concentration of the hydrogel solution at different temperatures was explored. Then, the relationship between the contact angle and the vascular wall thickness was analyzed by self-developed 3D bioprinting technology so as to achieve the purpose of printing blood vessels with different wall thicknesses by adjusting the contact angle.

## 2. Results

### 2.1. Contact Angle Measurement

The orthogonal experiment was designed using Design-Express software. According to the orthogonal experiment table, different contact angles were measured, and the results are shown in Table 1 (*n* = 16).

**Figure 1:**
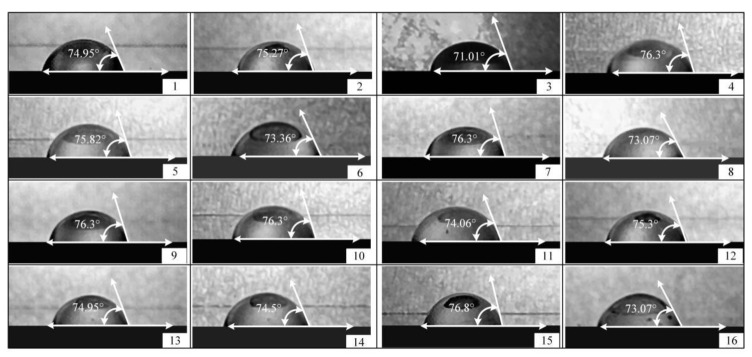
Contact angles of mixed hydrogels on stainless steel plates at different solution temperatures, gelatin concentrations, and alginate concentrations (*n* = 16). When the concentration of gelatin was 6 wt%, the concentration of sodium alginate was 7 wt%, and the solution temperature was 65 °C; the contact angle of the mixed hydrogel was the smallest. The minimum value is 71.01° (Groups 3). The contact angles of the mixed hydrogels were maximal when the gelatin concentration was 7 wt%, the sodium alginate concentration was 8wt%, and the solution temperature was 45 °C. The maximum is 76.8° (Groups 15).

Figure 1 describes the contact angles of mixed hydrogels on stainless steel plates at different solution temperatures, gelatin concentrations, and alginate concentrations (*n* = 16). The contact angles of the mixed hydrogels were changed by regulating the solution temperature, gelatin concentration, and alginate concentration. It was found that the difference between the maximum and minimum of the contact angle was 5.76° in 16 groups of tests, which also indicated that the temperature, gelatin concentration, and alginate concentration of the mixed hydrogels could have an effect on the contact angle of the mixed hydrogels.

#### 2.1.1. Range Analysis of the Experiment Data

In order to determine the degree of influence of each temperature and hydrogel solution concentration on the contact angle, the orthogonal experimental method was used to process the experimental data. The results of the extreme value analysis are shown in Table 2, where K is the sum of the experimental results for each factor level, k is the average of each factor level, and R is the range under this level.

The analysis results of the above experiments showed that temperature had the greatest effect on the variation of the contact angle, followed by the concentration of sodium alginate and, finally, the concentration of gelatin.

#### 2.1.2. Variance Analysis of Experiment Data

Because of the deficiency of direct analysis, variance analysis was used to optimize the experimental results; the experimental results were calculated and analyzed by analysis of variance (ANOVA). The analysis results are shown in Table 3.

The results of the one-way ANOVA showed that gelatin concentration, sodium alginate concentration, and the temperature of the mixed hydrogel solution all had significant effects on the contact angle, and the contact angle varied with the levels of each factor.

#### 2.1.3. Response Surface Analysis of the Experiment Data

**Figure 2:**
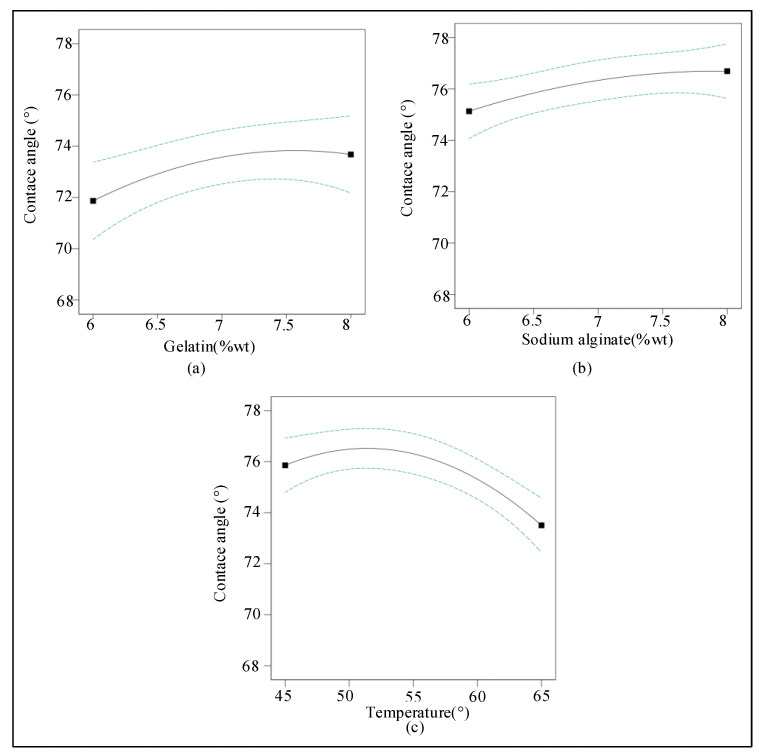
Effects of gelatin, sodium alginate, and temperature on hydrogel contact angles (*n* = 16). (**a**) The relationship between gelatin concentration (6–8 wt%) and contact angle (68–78°). (**b**) The relationship between sodium alginate concentration (6–8 wt%) and contact angle (68–78°). (**c**) The relationship between the temperature of the mixed hydrogel (45–65 °C) and the contact angle (68–78°).

The influence of a single factor on the contact angle is shown in Figure 2. It can be seen that the contact angle of the mixed hydrogel is proportional to the concentration of the gelatin (Figure 2a) and the concentration of the sodium alginate (Figure 2b). As the gelatin and sodium alginate solutions became thicker, the contact angle gradually increased. The reason for this phenomenon was that the increase in gelatin concentration and alginate concentration increases the viscosity of the mixed hydrogel, and the surface tension of the mixed hydrogel changes and increases the contact angle. With the increase in temperature, the contact angle of the mixed hydrogel increased first, reached the maximum when the temperature reached 52 °C, and then gradually decreased as the temperature continued to rise (Figure 2c). The reason for this phenomenon was that the viscosity of the gelatin solution decreased gradually with the increase of temperature in the mixed hydrogel, but the viscosity of the sodium alginate solution increased first and then decreased with the increase of temperature. Therefore, the contact angle of the mixed hydrogel solution increased first and then decreased with the change of viscosity.

**Figure 3:**
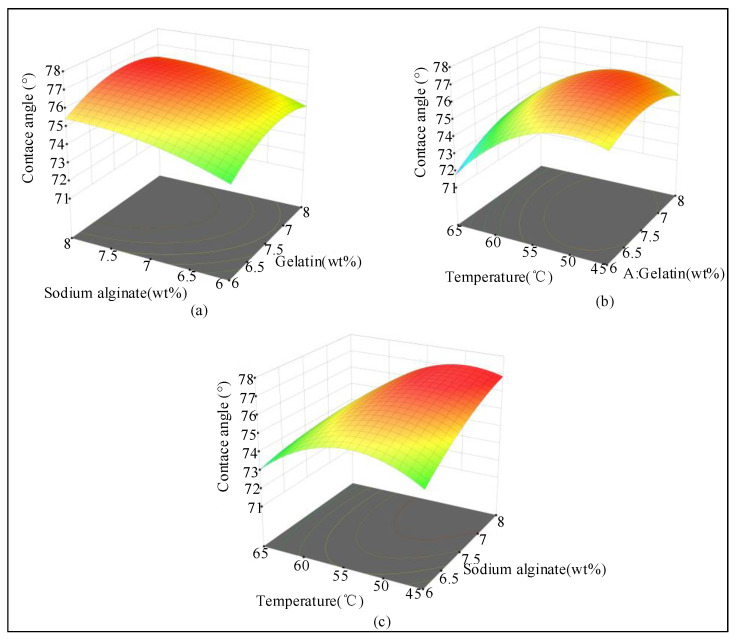
Influence of factor interaction on the contact angle (*n* = 16). (**a**) Effects of alginate concentration and gelatin concentration on the contact angle of the mixed hydrogel. (**b**) Effects of temperature and gelatin concentration on the contact angle of the mixed hydrogels. (**c**) Effects of temperature and alginate concentration on the contact angles of the mixed hydrogels. Temperature/alginate had the greatest effect on the contact angle, followed by temperature/gelatin, and, finally, gelatin/alginate.

According to the analysis shown in Figure 3, the interaction term (gelatin/alginate) had an effect on the contact angle. The interaction term (gelatin/temperature) and the interaction term (alginate/temperature) had a significant effect on the contact angle and increased first and then decreased with the increasing temperature.

### 2.2. Gradient Line Analysis

**Figure 4:**
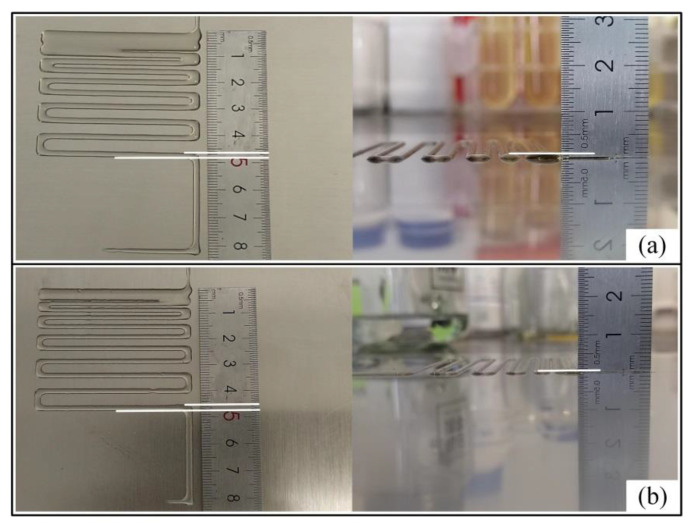
Two groups of gradient lines printed at different concentrations and temperatures. Comparison between the width and height of the gradient line filaments printed from gelatin/alginate/temperature (8–8 wt–55 °C) mixed hydrogel and gelatin/alginate/temperature (7–7 wt%–65 °C) mixed hydrogel. The height and width of the gradient lines were clearly different between the two groups. (**a**) The contact angle of the hybrid hydrogel was 76°, the width of the printing filament was about 2 mm, and the height was about 0.4 mm. (**b**) The contact angle of the mixed hydrogel was 73.5°, the width of the printed filament was about 2.5 mm, and the height was about 0.2 mm. As the hydrogel contact angle decreased, the printed filaments gradually became wider, and the height gradually decreased.

Due to the influence of the contact angle, the line width of hybrid hydrogels is different at different temperatures. The material accumulation caused by the same line spacing of hybrid hydrogels under different conditions also causes different forming heights. Figure 4 depicts the difference of the fusion spacing and printing molding height in (8–8 wt%–55 °C) the gelatin/sodium alginate mixed hydrogel (contact angle 76°) and (6–7 wt%–55 °C) the gelatin/sodium alginate mixed hydrogel (contact angle 73.5°).

It can be seen from Figure 4 that when the printed line spacing is relatively large, the line width is stable, and the line spacing can be clearly separated between the lines. As the line spacing decreases, the integration degree between the lines and the line spacing gradually increases, and the printed line will be deeply integrated into a closed state. Observing the lateral height, after the fusion of the printed lines, the height of the hydrogel gradually increased, and the spacing continued to decrease, causing an excessive material accumulation. In order to ensure the smooth progress of subsequent blood vessel printing and molding experiments, the second set of fusion line spacing among the gradient line spacing was selected as the printing distance according to the fusion between the mixed hydrogel filaments and the obtained height.

### 2.3. Analysis of Vessel Wall Thickness

**Figure 5:**
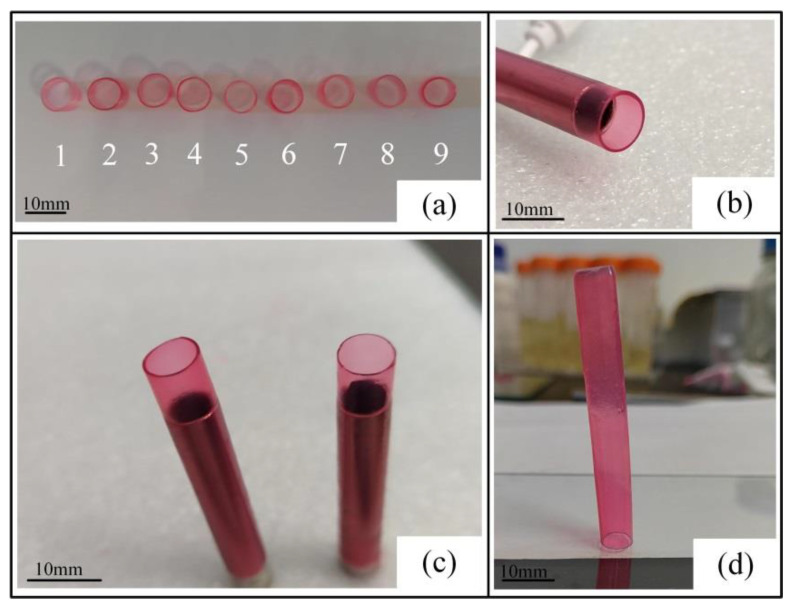
Vessel-like structure (*n* = 9). (**a**) With the increase of the hydrogel contact angle, the blood vessel wall thickness gradually became thicker, and the color became darker. The order of vessel wall from thick to thin was 6, 9, 4, 2, 7, 3, 8, 5, and 1, which was consistent with the measured hydrogel contact angle change. (**b**) Side view of vessel structure; the printed vessel has a good molding effect, and the vessel wall is symmetrical, which can be easily pulled out of the spiral rod. (**c**) Comparison between vessel wall thickness of 0.48 mm (contact angle of 76°) and vessel wall thickness of 0.39 mm (contact angle of 73.36°). The difference between the thickness can be clearly seen from the vascular section, and the vascular wall becomes thinner with the decrease of the contact angle. The larger the difference in the hydrogel contact angle, the more obvious the difference in the vessel wall thickness. (**d**) The artificial blood vessel, which is placed vertically on the glass plate after leaving the screw rod, has good elastic mechanical support performance.

The cross-linked artificial blood vessels were stained in red ink, and the thickness of the vessel wall was measured after removal. There were significant differences in the thickness of the artificial blood vessels printed under different conditions. The artificial blood vessels printed under different conditions are shown in Figure 5a.

The thickness of the vessel wall changed with the temperature and solution concentration. The wall thickness of the vessel was made easier to visualize by staining; the thicker the wall, the darker the color presented. As shown in Figure 5b,c, the external wall of the nine groups of blood vessels printed in this test was smooth, with a uniform shape and good elastic mechanical support performance. The 80 mm artificial blood vessel with a diameter of 8 mm can still be placed vertically on the surface of the glass plate after it is detached from the screw rod (Figure 5d).

**Figure 6:**
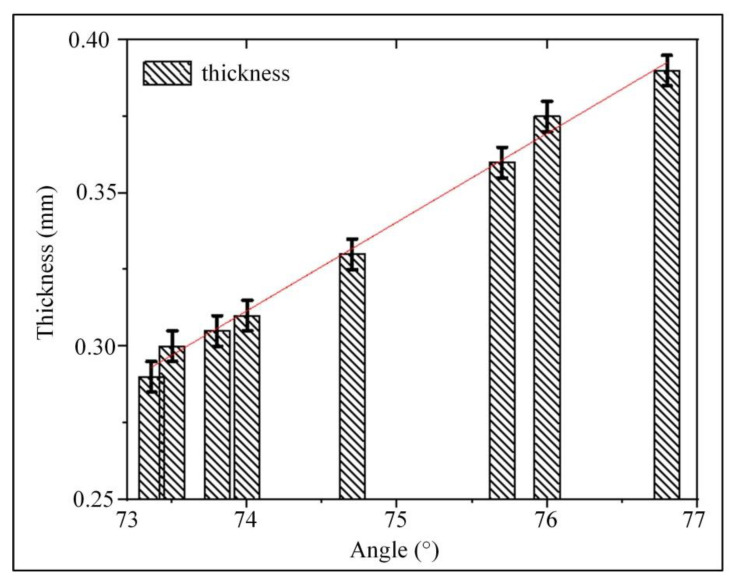
The relationship between the vessel wall thickness and contact angle. The resulting data were fitted using the least squares method to obtain the relationship between vessel wall thickness and contact angle (*n* = 9) The thickness of the vessel wall was proportional to the contact angle, and with the increase of the contact angle, the thickness of the vessel wall gradually became thicker.

According to Figure 6, the relationship between the contact angle and the wall thickness could be more intuitively reflected (*n* = 9). With the increase of the contact angle, the wall thickness of the artificial blood vessel gradually became thicker. When the contact angle was 73.36°, the wall thickness of the printed vessel was 0.29 mm. When the contact angle was 76°, the wall thickness gradually increased with the increase of the contact angle, reaching 0.48 mm. It can be demonstrated that there is a clear relationship between the vessel wall thickness and the contact angle of spiral-printed manufactured artificial blood vessels. The experimental results confirmed that changing the contact angle of the material can effectively control the wall thickness of the vascular structure.

## 3. Discussion

Many researchers [26,27] have proved that 3D printing of artificial blood vessels is expected to become an effective method for the treatment of cardiovascular and cerebrovascular diseases. However, the current 3D printing technology of artificial blood vessels has not formed a unified and complete process, resulting in the poor controllability of artificial blood vessel molding, which seriously affects the quality of the vascular structure molding [4].

The current methods of 3D bioprinting blood vessels can be divided into laser bioprinting, coaxial extrusion printing, non-supported blood vessel printing, and supported blood vessel printing. Laser bioprinting [28] has high accuracy, but it is not conducive to the survival of cells in bioink under light irradiation. Coaxial extrusion printing [29] is greatly affected by the printing speed and pressure, resulting in an uncontrollable vessel caliber, and there are fewer sacrificed-template materials to choose from. Layer-by-layer printing [30], without support, can print different shapes of blood vessels, but the resulting surface has poor quality and poor mechanical properties. Supported printing [24] can ensure the inner diameter of blood vessels. The printed blood vessels have a smooth surface and high molding quality, but the total size of the blood vessels cannot be guaranteed due to the influence of vessel wall thickness. Therefore, this paper attempts to explore a support printing method that can control the blood vessel wall thickness.

The surface contact angle can reflect the degree of diffusion of the liquid on the solid surface, and the greater the degree of diffusion, the smaller the contact angle. We speculated that the height of the droplets generated on the solid surface would decrease along with the smaller contact angle. Therefore, we tried to find a way to control the size of the contact angle and demonstrate the relationship between the height of the droplet on the solid surface and the contact angle. Thus, the controllability of the vessel wall thickness can be realized.

Firstly, the relationship between the contact angle and the concentration and temperature of the solution was explored. In terms of experimental concentration selection, after several attempts, it was confirmed that the printing effect was better when the concentration of gelatin was between 5–9% wt, and the molding effect was better when the concentration of sodium alginate was higher than 5% wt. Finally, gelatin concentrations between 6–8% wt and sodium alginate concentrations between 6–8% wt were selected to construct the vascular structure. This is similar to the result obtained by Giuseppe [31]. After measuring the contact angle, Design-Express software was used for analysis. It was concluded that the temperature and concentration of the mixed hydrogel solution had an effect on the contact angle. The contact angle of the hydrogel increased first and then decreased with the increase of temperature and gradually increased with the increase of the solution concentration.

Then, angiogenesis was studied based on a self-developed 3D printer. The printer was equipped with a temperature control system, multi-nozzle extrusion system, and extrusion pressure control system, which can realize multi-nozzle extrusion at a variety of temperatures and different pressures. In the gradient line printing experiment, the print height and line width gradually became taller and thinner as the contact angle increased. This was consistent with the previous conjecture that the droplet height would decrease at the same time that the contact angle decreased. Considering that the hydrogel is subjected to the centrifugal force of helix rod rotation during helical printing, the width of the line printed by the rotation will be slightly narrower than that printed by the gradient line. In order to ensure the success of the subsequent blood vessel printing experiments, we selected the second set of fusion line spacing in each set of printed gradient lines as the blood vessel printing distance according to the fusion between the mixed hydrogel filaments.

Among the printed blood vessel structure, the printed blood vessels (*n* = 9) had smooth surfaces, and all had good elasticity and mechanical support properties. By measuring the thickness of the vessel wall, it was found that the thickness of the vessel wall gradually thickened with the increase of the contact angle.

In summary, this paper designed experiments to derive the variation law of the contact angle magnitude of mixed hydrogel materials at different temperatures and concentrations. Based on the self-developed 3D bioprinter, the relationship between the contact angle and the thickness of the vessel wall was deduced. Then, by adjusting the concentration and temperature of the bioink, the contact angle of the bioink can be changed to regulate the thickness of the vessel wall. Thus, the thickness of the vessel wall was adjusted, and the thickness of the vessel wall was controllable. It provides a theoretical and technical basis for the treatment of cardiovascular and cerebrovascular diseases.

## 4. Materials and Methodology

### 4.1. Materials

To ensure uniform mixing of hydrogels, gelatin (G6317, pigskin gelatin, purchased from Shanghai Macklin Biochemical Co. Ltd, Shanghai, China) was added to distilled water and stirred for 3 h at 1200 r/min in a 65 °C water bath using a magnetic stirring water bath (Changzhou Jintan Friend Instrument Co. Ltd, Jiangsu, China). Then, sodium alginate (S817372, viscosity 200 ± 20 mpa.s, purchased from Shanghai Macklin Biochemical Co. Ltd, Shanghai, China) was added to continued stirring, and gelatin/sodium alginate composite hydrogel solutions with different mass percentages were configured. When the concentration of gelatin was less than 4%, the solution molding effect was poor, the concentration was higher than 10%, easy to block the nozzle, and the concentration of 6% to 8% toughness and molding effect was the best. The viscosity changed little at lower alginate concentrations and increased when alginate concentrations were higher than 5%. The proportions of gelatin and sodium alginate configurations selected after comprehensive consideration are shown in Table 4.

**Figure 7:**
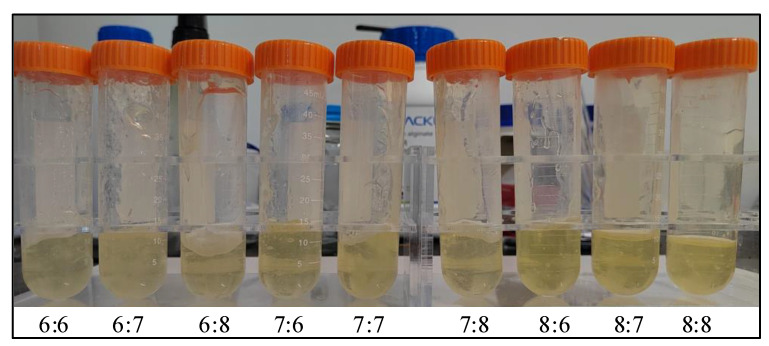
Gelatin/alginate solutions at different concentrations (gelatin: sodium alginate, *n* = 9). The color of the mixed hydrogel solution gradually became darker, and the viscosity gradually increased as the overall concentration increased. When the alginate concentration was the same, the mixed hydrogel solution became darker with the increase of gelatin. At the same concentration of gelatin, the viscosity of the mixed aqueous solution gradually increased with the increase of sodium alginate.

After the sodium alginate and gelatin were completely dissolved, the mixed hydrogel was centrifuged at 3500 r/min for 15 min using a tabletop high-speed centrifuge (purchased from Shanghai Macklin Biochemical Co. Ltd, Shanghai, China) to ensure complete removal of air bubbles and, finally, frozen in the refrigerator. Figure 7 shows the mixed hydrogels with different concentrations. The color of the mixed hydrogel and the gelatin body is light yellow; the higher the concentration of the mixed solution was, the darker the color, and the viscosity increased with the increase of concentration.

### 4.2. Methodology

#### 4.2.1. Contact Angle Experiment

(1) Contact angle

**Figure 8:**
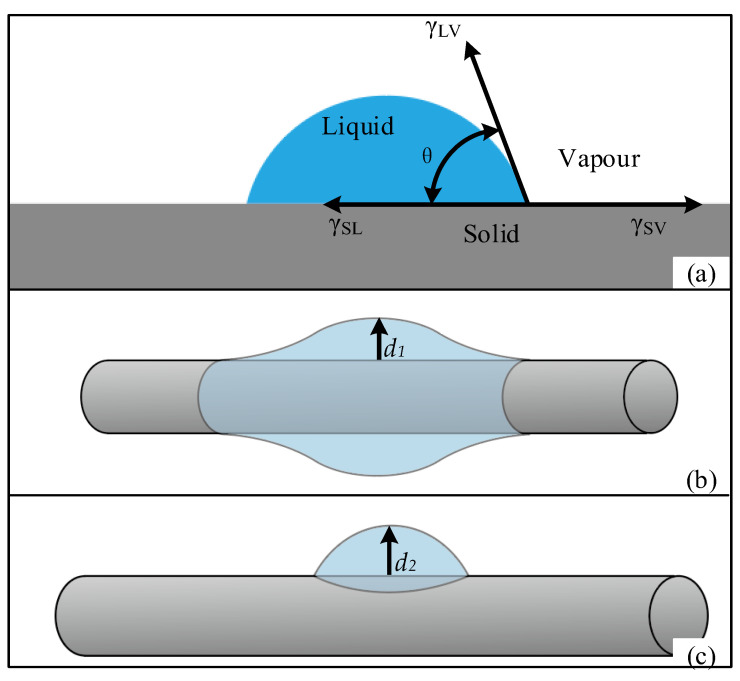
Contact angle. (**a**) The droplet formed on the solid plane and its contact angle θ. The two equilibrium states of the droplets formed on the surface of the cylinder are (**b**) barrel-type and (**c**) clam-shell. The droplets formed on the cylinder surface at heights *d*_1_ and *d*_2_, respectively.

The contact angle can intuitively reflect the degree of infiltration of a certain liquid into a solid. When liquid droplets of different viscosity are added to the solid surface, the droplets will form different shapes due to the difference in infiltration. As shown in Figure 8a, the angle between the vapor–liquid connection line and the solid–liquid connection line is called the contact angle, which is usually represented by θ. The contact angle of the droplet on an ideal smooth surface can be expressed by Young’s equation:

(1) $$\cos\theta =\frac{\Upsilon_{SV}-\Upsilon_{SL}}{\Upsilon_{LV}}$$

where θ is the contact angle, $\Upsilon_{SV}$ is the surface tension of the solid and vapor, $\Upsilon_{SL}$ is the surface tension of the solid and liquid, and $\Upsilon_{LV}$ is the surface tension of the liquid and vapor.

As shown in Figure 8b,c, when the solid surface shape is cylindrical, the droplet contour expands along the curve of the cylindrical surface, and the three-phase interface is not straight so that the droplet equilibrium state is barrel-type or clam-shell, and the contact angle is different so that the liquid height formed on the cylindrical surface is also different.

(2) Contact angle analysis

The mixed hydrogel removed from the refrigerator was heated to 45 °C to 65 °C, and 10 uL of each hydrogel was dropped onto a stainless steel plate with a liquid injection needle (Changsha Bega Scientific Instruments Co. Ltd, Hunan, China), and the contact angle was measured after standing for 30 s.

Considering that the temperature and solution concentration will affect the contact angle, this experiment was designed as a three-factor and three-level experiment, and the specific factor levels are shown in Table 5.

#### 4.2.2. 3D Bioprinting

(1) Multi-nozzle 3D bioprinter

**Figure 9:**
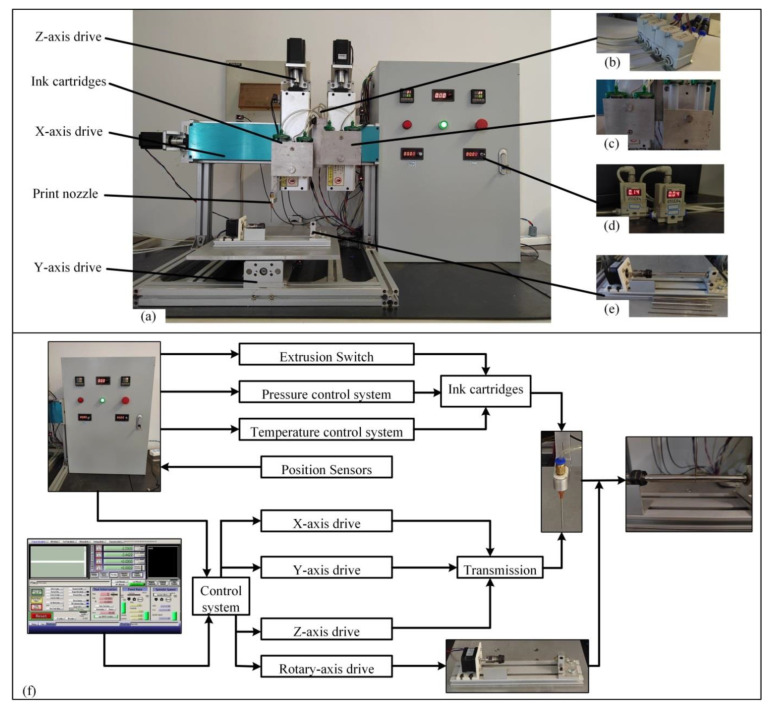
Self-developed 3D bioprinter. (**a**) 3D bioprinter and I/O control cabinet. (**b**) Multi-nozzle extrusion system: four ventilation valves were connected to each of the four cartridges to provide pressure for the extrusion of bioink from the cartridge. (**c**) Rotary forming system: according to the diameter of the replacement screw rod, different inner diameters of artificial blood vessels can be printed. (**d**) Temperature control system: the cartridge is embedded in a thermostat plate, and the thermostat plate temperature can be adjusted by controlling the thermostat on the I/O control cabinet. (**e**) Extrusion pressure control system: adjust the extrusion pressure, and the pressure values shown in the picture are 0.14 Mpa and 0.04 Mpa. (**f**) Workflow of 3D bioprinter: the artificial blood vessel was printed by the main body of the printer, I/O control cabinet, and MACH3 software.

The 3D printer used in this experiment was a self-developed multi-nozzle 3D biological printer based on a MACH3 control board (Figure 9a). The printer was composed of a motion control system, multi-nozzle extrusion system, and rotary forming system, which can simultaneously control the operation of multiple nozzles at the same time and is equipped with a temperature control system and extrusion pressure control system. It can make the biological ink extrusion at a constant temperature during the printing process, which meets the needs of this experiment.

The motion control system consists of an *x*-axis, *y*-axis, *z*1-axis, *z*2-axis, and *c*-axis. The printing nozzles are fixed on the two *z*1-axis and *z*2-axis, respectively, and the printing platform is fixed on the *y*-axis. When the bio-printer works, the *x*-axis and *z*-axis drive the nozzle movement, and the *y*-axis drives the printing platform movement to realize the space movement of the printing nozzle on the printing platform. The multi-nozzle extrusion system (Figure 9b) consists of four independent ventilation valves, each of which controls the on and off pressure of the four cartridges. The rotary forming system (Figure 9c) is driven by a stepper motor to rotate the fixture. The clamp can hold the screw rod, and the end of the screw rod is fixed with a rotary thimble to ensure the coaxiality of the blood vessels when printing. The rotary molding system works with the motion control system to print artificial blood vessels of different diameters by replacing different diameters of screw rods. The temperature control system (Figure 9d) consists of a constant temperature plate and a thermostat in which the ink cartridge is embedded. The heating element and temperature sensor in the thermostat plate are connected to the thermostat, which can adjust the real-time temperature at any time so that the biological ink in the cartridge can always maintain the temperature needed for the experiment. The extrusion pressure control system (Figure 9e) controls and adjusts the real-time pressure through an external switch, which can provide a different extrusion pressure for the two groups of nozzles.

Figure 9f shows the control system composition of the 3D bioprinter. The user mainly controls the 3D bioprinter by controlling the I/O module and the external computer. The I/O module mainly adjusts the extrusion switch, pressure control system, and temperature control system to control the temperature and extrusion pressure of the cartridge. At the same time, the position sensor feeds back the real-time position to the control system through the I/O module. The external computer input program with the control system realizes the nozzle’s *x* direction, *y* direction, *z* direction movement, and *c*-axis rotation. Under the joint action of the control system and I/O module, when the rotation axis and the nozzle move, the bioink is extruded at the set temperature and pressure. Finally, the printing of artificial blood vessels is realized.

(2) Gradient line printing

**Figure 10:**
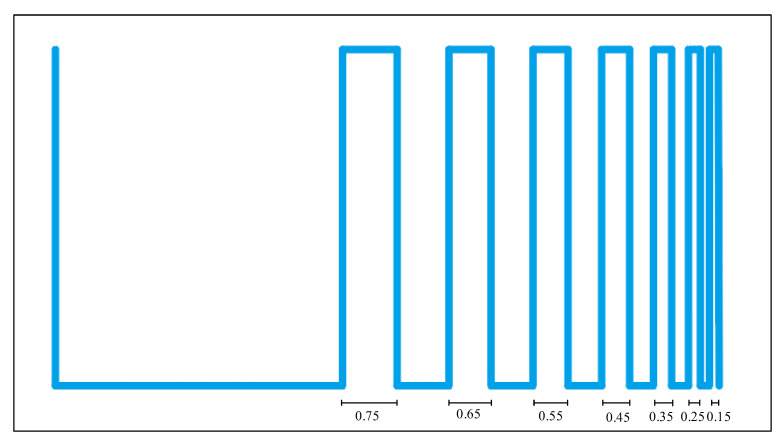
Print of the gradient line trajectory.

In order to evaluate the filaments fusion effect of hydrogels, the printing path of the filaments fusion experiment with line spacing was designed (Figure 10). The line structure with increasing line spacing was printed to observe the fusion status between adjacent lines at different line spacing.

The hydrogel was liquefied by heating to 45 °C and poured into a 3D bioprinter cartridge. Print experiments were performed using the cartridge temperature control system, adjusted to the specified temperature (*n* = 9). The extrusion pressure control system was used to adjust the extrusion pressure between 0.04 and 0.08 MPa to make the hydrogel extrusion uniform. A nozzle with a diameter of 2 mm was used, the printing height was set to 0.9 mm, and the feeding speed was 6 mm/s. Gradient line printing tests were performed on stainless steel plates. The gradient line was 50 mm in length, and the line spacing was reduced from 0.75 mm to 0.15 mm, and the reduction was 0.05 mm each time. After printing, the mixture was allowed to stand for 30 s, and the spacing and formation height of the gelatin/sodium alginate mixed hydrogel filaments were measured at different temperatures and concentrations.

(3) Artificial blood vessel printing

**Figure 11:**
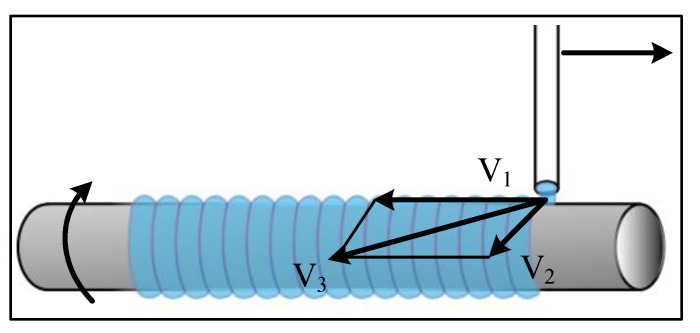
Spiral printed feed velocity vector plot.

The artificial blood vessel printed at this time was printed by extrusion rotation, as shown in Figure 11. The extrusion material was taken as a reference, and the printing speed, *V*_3_, is the synthesis speed of the *x*-axis feed speed, *V*_1_, and the rotation axis speed, *V*_2_. The speed formula is as follows:

(2) $$\vec{V_{3}}=\vec{V_{1}}+\vec{V_{2}}$$

The stainless steel tube used for printing was 8mm in diameter and made of the same quality as the stainless steel sheet used for printing the gradient lines. The optimal spacing between the fusion lines of the hydrogels in each group was selected in the gradient line printing test. and the actual printing speed was calculated according to Equation (2) to ensure the effect. Other parameters were consistent with those of the printed gradient line. After printing, artificial blood vessels were cross-linked in a 5 wt% CaCl_2_ solution, and the blood vessel wall thickness of each group was measured after cross-linking.

## 5. Conclusions

The 3D bioprinting method of small-caliber vascular structures is expected to achieve vascular structure regeneration, which has become an effective measure to solve major vascular diseases such as heart and peripheral vascular diseases. This study established the feasibility of a self-developed 3D bioprinter to carry out research on the controllable molding of the vascular structure. The 3D bioprinter with a multi-nozzle function can achieve the rapid printing of vascular structures. Orthogonal experiments were designed to verify the controllable law of the material infiltration angle and then to confirm the influence mechanism of the infiltration angle and vessel wall thickness, which can provide a theoretical basis for the further study of small diameter artificial blood vessels. The subsequent steps will be to optimize the material properties, improve the microstructure of the material contact surface, develop a protocols scheme of the controllable molding of the vascular structure to ensure the mechanical properties of the vascular structure, and remodel the artificial blood vessel structure in the treatment of major vascular diseases such as cardiac and peripheral vascular diseases.

## Figures and Tables

**Table 1:** Orthogonal experiment results (*n* = 16).

Orthogonal Experiment	Gelatin (wt%)	Sodium Alginate (wt%)	Temperature (°C)	Contact Angle (°)
1	8	6	55	74.95
2	6	8	55	75.27
3	6	7	65	71.01
4	7	7	55	76.3
5	8	7	45	75.82
6	8	7	65	73.36
7	7	7	55	76.3
8	7	6	65	73.07
9	7	7	55	76.3
10	7	7	55	76.3
11	7	8	65	74.06
12	6	7	45	75.3
13	8	8	55	74.95
14	6	6	55	74.5
15	7	8	45	76.8
16	7	6	45	73.07

**Table 2:** Extreme analysis.

Experiment Results	Gelatin	Sodium Alginate	Temperature
K1	296.08	295.59	300.99
K2	678.50	676.99	681.17
K3	299.08	301.08	291.5
k1	74.02	73.90	75.25
k2	75.29	75.22	75.68
k3	74.77	75.27	72.87
Range R	1.36	1.37	2.81
Major factor → Minor factor	Temperature → Sodium alginate → Gelatin		

**Table 3:** Variance analysis of experiment data.

Variance Analysis	Variance Source	Freedom	Square Sum	Mean Square	F Value	*p* Value	Significance
1	A-Gelatin	1	1.67	1.67	2.98	0.1282	Significant
2	B-Sodium alginate	1	4.71	4.71	8.42	0.0229	Extremely significant
3	C-Temperature	1	11.26	11.26	20.12	0.0028	Extremely significant
4	AB	1	0.0036	0.0036	0.0064	0.9383	Non-significant
5	AC	1	0.8372	0.8372	1.5	0.2608	Significant
6	BC	1	1.88	1.88	3.35	0.1097	Significant
7	A^2^	1	2.69	2.69	4.8	0.0646	Significant
8	B^2^	1	0.7472	0.7472	1.34	0.2858	Significant
9	C^2^	1	11.17	11.17	19.96	0.0029	Extremely significant

**Table 4:** Material parameters (*n* = 9).

Sample Serial Number	Quality Percentage (wt%)	
	Gelatin	Sodium Alginate
1	6	6
2	6	7
3	6	8
4	7	6
5	7	7
6	7	8
7	8	6
8	8	7
9	8	8

**Table 5:** Factors and levels of orthogonal text (*n* = 9).

Sample Serial Number	Gelatin (wt%)	Sodium Alginate (wt%)	Temperature (°C)
1	6	6	45
2	6	7	55
3	6	8	65
4	7	6	55
5	7	7	65
6	7	8	45
7	8	6	65
8	8	7	45
9	8	8	55

## Data Availability

All data generated or analyzed during this study are included in this published article.
